# Transcriptional Network Analysis Reveals the Role of miR-223-5p During Diabetic Corneal Epithelial Regeneration

**DOI:** 10.3389/fmolb.2021.737472

**Published:** 2021-08-26

**Authors:** Yuan Zhang, Shengqian Dou, Xia Qi, Zhenzhen Zhang, Yujie Qiao, Yani Wang, Jin Xie, Hui Jiang, Bin Zhang, Qingjun Zhou, Qun Wang, Lixin Xie

**Affiliations:** ^1^ State Key Laboratory Cultivation Base, Shandong Provincial Key Laboratory of Ophthalmology, Shandong Eye Institute, Shandong First Medical University and Shandong Academy of Medical Sciences, Qingdao, China; ^2^ Department of Ophthalmology, Zhongnan Hospital of Wuhan University, Wuhan, China; ^3^ Eye Center, Renmin Hospital of Wuhan University, Wuhan, China; ^4^ Qingdao Eye Hospital of Shandong First Medical University, Qingdao, China; ^5^ Medical College, Qingdao University, Qingdao, China

**Keywords:** diabetic keratopathy, wound healing, high-throughput sequencing, miR-223-5p, *Hpgds*

## Abstract

Diabetes mellitus (DM) is a complex metabolic disorder. Long-term hyperglycemia may induce diabetic keratopathy (DK), which is mainly characterized by delayed corneal epithelial regeneration. MicroRNAs (miRNAs) have been reported to play regulatory roles during tissue regeneration. However, the molecular mechanism by which miRNAs influence epithelial regeneration in DK is largely unknown. In this study, we performed miRNA and mRNA sequencing of regenerative corneal epithelium tissue from streptozotocin-induced type 1 diabetic (T1DM) and wild-type mice to screen for differentially expressed miRNAs and mRNAs. Based on regulatory network analysis, miR-223-5p was selected for subsequent experiments and *Hpgds* was then identified as a direct target gene. MiR-223-5p downregulation significantly promoted diabetic corneal epithelial wound healing and nerve regeneration. However, the beneficial effects of miR-223-5p inhibition were abolished by an Hpgds inhibitor. Furthermore, mechanistic studies demonstrated that miR-223-5p suppression ameliorated inflammation and enhanced cell proliferation signaling in DK. Taken together, our findings revealed that the regulatory role of miR-223-5p in diabetic corneal epithelial and nerve regeneration by mediating inflammatory processes and cell proliferation signaling. And silencing miR-223-5p may contribute to the development of potential therapeutic strategies for DK.

## Introduction

Diabetes mellitus (DM), a widespread health concern and is the most common metabolic disorder. Diabetic keratopathy (DK), a major chronic complication of DM, affecting up to two-thirds of diabetic patients (Barsegian et al., 2018; Han et al., 2019). Persistent hyperglycemia can cause progressive damage of corneal structures and functions, that may lead to the loss of corneal sensitivity, basement membrane abnormality, the increase of stromal thickness, and the decrease of corneal endothelial density (Friend et al., 1982; Urban et al., 2013; Gao et al., 2016; Kumar et al., 2018). Corneal epithelium is the initial barrier between the external environment and the eye, functioning in an important defensive role (Zhu et al., 2019a). The delayed corneal re-epithelialization is a common complication of DK (Bu et al., 2019; Li et al., 2020a; Wang et al., 2020a; Li et al., 2020b). In the past few years, our group and other scholars have uncovered a set of molecules playing vital roles in diabetic corneal re-epithelialization, including phosphatase and tensin homologue (PTEN), substance P (SP), mesencephalic astrocyte-derived neurotrophic factor (MANF), leucine-rich α-2-glycoprotein-1 (LRG1), silent mating type information regulation 2 homolog 3 (SIRT3) and semaphorin (SEMA) 3C. (Yang et al., 2014; Lee et al., 2019; Hu et al., 2019b; Li et al., 2020a; Wang et al., 2020a; Li et al., 2020b). However, the pathogenesis of DK remains to be clarified, and the treatment of DK is still an ongoing clinical challenge.

To enrich the regulatory factors and underlying mechanisms involved in DK, we focused on epigenetic regulator-miRNAs. MiRNAs are endogenous single-stranded non-coding RNAs containing about 23 nucleotides, and are known to act as key post-transcriptional regulators by promoting mRNA decay or suppressing translational processes (He and Hannon, 2004; Findeiss et al., 2021). MiRNA-targeted therapeutics have reached the stage of clinical trial development, including treatment with miRNA mimics or anti-miRNAs (Rupaimoole and Slack, 2017; Kramerov et al., 2021). It was reported that microRNAs (miRNAs)-mediated regulation of gene expression are involved in the corneal re-epithelialization and nerve repair through different mechanisms (Funari et al., 2013; Gao et al., 2015; Wang et al., 2016; Hu et al., 2019a; Hu et al., 2020). MiRNA-204-5p was shown to participate in delaying epithelium cell traversal biological processes (Gao et al., 2015). In addition, miR-146a and miR-424 were reported to be upregulated in diabetic corneas and contribute to delaying wound closure in human corneal epithelial cells (Funari et al., 2013). Nonetheless, current studies have not comprehensively or fully revealed the miRNA-based regulatory networks and underlying mechanisms involved in delayed diabetic corneal epithelial regeneration.

A combination of high-throughput technologies and bioinformatic analysis could be used to efficiently screen for novel biomarkers and therapeutic targets for diseases. In the placentas of gestational diabetes mellitus (GDM) patients, 281 mRNAs and 32 miRNAs were screened out by RNA sequencing. miR-138-5p, selected from the bioinformatics analysis, was further found to play a role in the pathology of GDM (Ding et al., 2018). Further, the emerging role of miR-221-3p in diabetic skin wound healing was identified by high-throughput sequencing, animal experiments and bioinformatics analysis (Xu J. et al., 2020). In terms of DK, miR-10b was found to be upregulated in the limbus *versus* central cornea and in the diabetic *versus* normal limbus by deep sequencing analysis, and may mediate corneal epithelial homeostasis and stem cell function (Kulkarni et al., 2017).

In this study, miRNA and mRNA sequencing were simultaneously performed on samples obtained from the diabetic and normal healing corneal epithelium. After network analysis and application of stringent screening criteria, the miR-223-5p/*Hpgds* axis was identified. Further functional assays revealed that suppressing miR-223-5p could promote diabetic corneal epithelial and nerve regeneration by regulating inflammatory processes and cell proliferation signaling. Overall, this study provides significant information to improve the understanding of DK pathogenesis, and might deliver potential diagnostic biomarkers or therapeutic targets for interventions in DK.

## Materials and Methods

### Animals

C57BL/6 mice (6–8 weeks old, male) were provided by the Beijing Vital River Laboratory Animal Technology Co., Ltd. (Beijing, China). The animals were housed at the animal center of Shandong Eye Institute according to the Principles of Laboratory Animal Care. The use of animals in this study complied with the Committee guidelines of the Shandong Eye Institute and the Association for Research in Vision and Ophthalmology Statement for the Use of Animals in Ophthalmic and Vision Research. Type 1 diabetic mice (T1DM) were induced by intraperitoneal injection of low-dose streptozotocin (STZ) 50 mg/kg (Sigma-Aldrich, St. Louis, MO, United States) for 5 consecutive days as previously described (Wang et al., 2020a; Li et al., 2020b). Tail-vein blood glucose concentrations and body weight of animals were determined (*n* = 12 per group). In the current study, diabetic mice were selected after 16 weeks of final STZ injection, with a blood glucose level above 25 mmol/L. For all animal experiments, only one eye was wounded in each mouse.

### Corneal Sensitivity Measurement

Corneal sensitivity measurements were performed with a Cochet-Bonnet esthesiometer (Luneau Ophtalmologie, Chartres Cedex, France) in unanesthetized mice according to the reports (Yang et al., 2014). The experiment started from the maximally extended length (6 cm) of a nylon monofilament and was shortened by 0.5 cm each measurement until a blink response occurred. The longest filament length resulting in a positive response was considered as the corneal sensitivity threshold. Each group includes 12 eyes. The measurement was performed at least four times per eye.

### Corneal Epithelial Debridement

Mice were anesthetized by an intraperitoneal injection of 0.6% sodium pentobarbital, followed by topical application of 0.5% proparacaine. The corneal epithelium (2.5-mm in diameter) was removed with an Algerbrush II rust ring remover (Alger Co., Lago Vista, TX, United States) as described in previous studies (Li et al., 2020a; Wang et al., 2020a). To evaluate corneal wound healing, the residual corneal epithelium defects were visualized at 0, 12, 24, and 36 h after injury by staining with 0.25% fluorescein sodium and were then photographed under a slit lamp microscope. The defect area was calculated using ImageJ software.

### RNA Sequencing and Data Processing

To perform RNA sequencing, samples of regenerative corneal epithelium including the limbus and part of the regenerating central cornea from 24 corneas of 24 mice were harvested 24 h after debridement. These samples were pooled into six groups (normal mice with corneal injury groups 1–3 and diabetic mice with corneal injury groups 4–6, each group included corneal epithelium from four mice). Next, the epithelium was disrupted in TRIzol reagent (Invitrogen, United States) immediately and stored at −80°C until further experiments could be performed. The RNA obtained was run on agarose gels to measure degradation and contamination. RNA quantity and quality were also estimated using the NanoDrop ND-1000 system. Complementary DNA (cDNA) libraries were prepared using NEB Multiplex Small RNA Library Prep Set (Illumina, United States) for miRNA and KAPA Stranded RNA-Seq Library Prep Kit (Illumina, United States) for mRNA. Then, the quality of the amplified libraries was verified with a 2,100 Bioanalyzer (Agilent, United States). Sequencing of high-quality miRNA and mRNA was carried out using the Illumina NextSeq 500 (Illumina, United States) and the Illumina NovaSeq 6000 (Illumina, United States), respectively.

Screening for differentially expressed miRNAs (DEmiRNAs) and differentially expressed mRNAs (DEmRNAs) were performed on paired samples. To screen for DEmiRNAs, an edgeR analytic tool was applied. A *p*-value of <0.05, group counts per million (CPM) ≥1, and |log2 fold change (FC)| ≥0.585 were set as the cut-off criteria to identify DEmiRNA. For mRNA sequencing data, DEmRNAs were analyzed using ballgown. All mRNAs with a *p*-value of <0.05, fragments per kilo base per million mapped reads (FPKM) ≥0.5, and |log2FC| ≥0.585 were viewed as DEmRNAs. Volcano plots were developed to map the differentially expressed genes.

### Functional Annotations

Gene ontology (GO) incorporates three ontologies—molecular function (MF), cellular components (CC), and biological processes (BP) of genes. To investigate the potential functions of DEmRNAs, we conducted GO analysis based on http://www.geneontology.org. A *p*-value of <0.05 was considered statistically significant.

### Construction of miRNA/mRNA Interaction Networks

To perform a specific miRNA interactome for diabetic corneal re-epithelialization, we used an integrated stepwise prioritization method. Targetscan is a database used for miRNA target prediction. Thus, target genes were first predicted using Targetscan. Inversely correlated FC of DEmRNA and DEmiRNA of the paired samples was the criterion considered to identify regulatory pairs. We explored the predicted target genes of decreased miRNAs and increased miRNAs from upregulated mRNAs and downregulated mRNAs, respectively. Cytoscape was used to visualize the miRNA/mRNA regulatory network. Furthermore, in order to obtain more credible targets, target genes in the intersection of Targetscan with a cumulative weighted context++ score and a total context++ score <−0.3, and miRDB with a score ≥70 were retained.

### Quantitative Real-Time Polymerase Chain Reaction Analysis

Total RNA (*n* = 4 per group), including miRNA, from regenerative corneal epithelium was extracted using TRIzol reagent (Invitrogen, United States). As for miRNAs, the high quality total RNAs were used as the template to obtain cDNA utilizing M-MuLV reverse transcriptase (Epicentre, United States). For mRNAs, cDNA synthesis was carried out using the SuperScriptTM III reverse transcriptase (Invitrogen, United States). Finally, quantitative real-time polymerase chain reaction analysis (qRT-PCR) was performed in triplicate using 2 × PCR master mix (Arraystar, United States). U6 was used as the endogenous control for normalizing the expression levels of miRNAs. The expression levels of mRNAs were normalized to that of β-actin. Primer sequences are listed in Tables 1, 2.

**TABLE 1:** miRNA stem-loop primers used for qRT-PCR.

Gene	Primer sequences
miR-223-5p	RT: 5′GTC​GTA​TCC​AGT​GCG​TGT​CGT​GGA​GTC​GGC​AAT​TGC​ACT​GGA​TAC​GAC​CAA​CTC​A3′
	GSP: 5′GGG​AGT​CGT​GTA​TTT​GAC​AAG​C3′
	R: 5′GTG​CGT​GTC​GTG​GAG​TCG3′
miR-212-3p	RT: 5′GTC​GTA​TCC​AGT​GCG​TGT​CGT​GGA​GTC​GGC​AAT​TGC​ACT​GGA​TAC​GAC​TGG​CCG3′
	GSP:5′GGGGGATAACAGTCTCCAGTCA3′
	R:5′GTGCGTGTCGTGGAGTCG3′
miR-210-5p	RT: 5′GTC​GTA​TCC​AGT​GCG​TGT​CGT​GGA​GTC​GGC​AAT​TGC​ACT​GGA​TAC​GAC​CAG​TGT​GC3′
	GSP:5′GAAAAGCCACTGCCCACC3′
	R:5′GTGCGTGTCGTGGAGTCG3′
U6	RT: 5′CGC​TTC​ACG​AAT​TTG​CGT​GTC​AT3′
	F: 5′GCT​TCG​GCA​GCA​CAT​ATA​CTA​AAA​T3′
	R: 5′CGC​TTC​ACG​AAT​TTG​CGT​GTC​AT3′

RT, Primer for reverse transcription; GSP, gene-specific primer; F, forward primer; R, reverse primer.

**TABLE 2:** mRNA primers used for qRT-PCR.

Gene	Forward primer	Reverse primer
β-actin	F:5′ GTA​CCA​CCA​TGT​ACC​CAG​GC3′	R :5′AAC​GCA​GCT​CAG​TAA​CAG​TCC3′
Hpgds	F:5′TGGTGGACACGCTGGATGAC3′	R:5′AGAAGGCGAGGTGCTTGATGT3′
Hmga2	F:5′AGCAAGAGCCAACCTGTGA3′	R:5′AGGCTTCTTCTGAACGACTTG3′
Msln	F:5′TGAAGTGCCAGGGCGTGTA3′	R:5′AACCTCCCTGACTGCCTTTTC3′
CD45	F:5′GGTTGTTCTGTGCCTTGTTCAA3′	R:5′ TGG​CGA​TGA​TGT​CAT​AGA​GGA​A 3′
TNF-α	F:5′ ATG​AGA​AGT​TCC​CAA​ATG​GC′	R:5′ CTC​CAC​TTG​GTG​GTT​TGC​TA 3′
IL-6	F:5′ ACC​ACT​CCC​AAC​AGA​CCT​GTC​T 3′	R:5′ CAG​ATT​GTT​TTC​TGC​AAG​TGC​AT3′
IL-1β	F:5′ CTT​TCC​CGT​GGA​CCT​TCC​A 3′	R:5′ CTC​GGA​GCC​TGT​AGT​GCA​GTT 3′

F, forward primer; R, reverse primer.

### Subconjunctival Injection of the Antagomir and Inhibitor

Subconjunctival injection is routinely used in clinical treatment and in experimental studies of ocular diseases (Wang et al., 2020a). Anesthetized mice were injected subconjunctivally with 10 μL solution per injection. miR-223-5p antagomir (20 μmol/L) from GenePharma (Shanghai, China), miRNA antagomir negative control (NC) (20 μmol/L) from GenePharma (Shanghai, China). HQL-79 (50 μg/ml) (Cayman, 10134), and vehicle (2% methanol) were injected subconjunctivally 24 h before and 0 h after injury. At 24 h after wounding, corneas were collected for qRT-PCR, western blot and immunofluorescence staining. On the fifth day after the corneal epithelial debridement, corneas were used for corneal whole-mount staining and corneal sensitivity measurement. In the untreated normal and diabetic mice, the right eyes were untreated after injury.

### Western Blotting

At 24 h after debridement, protein samples of the wounded corneas were harvested. For each group, six corneas form six mice were collected. Samples (20 μg) were run on SDS-PAGE and then transferred to a polyvinylidene fluoride (PVDF) membrane. Membranes were blocked with 5% bovine serum albumin (BSA) at room temperature for 2 h and then incubated at 4°C overnight with the primary antibodies against hematopoietic prostaglandin D synthase (Hpgds) (Cayman, 160013), phosphorylated protein kinase B (p-AKT) (Cell Signaling Technology, 4060), AKT (Cell Signaling Technology, 4691), phosphorylated signal transducer and activator of transcription-3 (p-STAT3) (Abcam, ab76315), STAT3 (Cell Signaling Technology, 4904), and β-Actin (Affinity, AF7018), followed by incubation with horseradish peroxidase-conjugated secondary antibodies.

### Luciferase Activity Assay

Mouse *Hpgds* 3′-UTR including the putative target site for miR-223-5p was amplified by PCR and inserted into the pMIR-REPORT (RiboBio). The mutation from a site of perfect complementarity was also created. HEK293T cells were co-cultured with wild-type or mutant reporter plasmid and co-transfected with miR-223-5p mimics or negative control (NC) and cultured for 48 h. Then the luciferase activity was measured.

### Immunofluorescence Staining

Immunofluorescence staining was performed as follows (Li et al., 2020a; Li et al., 2020b). Seven-micrometer-thick frozen tissues sections were prepared and were fixed by 4% paraformaldehyde for 15 min. For p-AKT (Cell Signaling Technology, 4060) and p-STAT3 (Abcam, ab76315) staining, samples were permeabilized with 0.1% Triton X-100 for 10 min, while staining CD45 (Invitrogen, 11-0451-81) did not require this process. Next, samples were blocked with 5% BSA, incubated with antibodies, and counterstained with 4′,6-diamidino-2-phenylindole (DAPI). The staining results were observed under a fluorescence microscope (Nikon, Tokyo, Japan).

### Whole-Mount Staining of Corneal Nerves

On day 5 after the corneal epithelial debridement, corneal whole-mount staining was performed. Eyeballs were fixed in Zamboni stationary liquid for 1 hour. After that, corneas were dissected around the scleral-limbal part and fixed in Zamboni stationary liquid for another hour. Fixed corneas were blocked by Tris-buffered saline (TBS) containing 0.1% Triton X-100, 2% goat serum, and 2% BSA followed by staining with Alexa Fluor 488 conjugated neuronal class III beta-tubulin mouse monoclonal antibody (Merck Millipore, AB15708A4) overnight at 4°C. Subsequently, the corneas were washed by phosphate buffered saline (PBS) and observed under a confocal microscope (Zeiss, Germany). The overall corneal nerve fiber content was calculated as the percentage of area positive for β-tubulin III staining determined with Image J software (Di et al., 2017; Lee et al., 2019; Li et al., 2020b).

### Statistical Analysis

All the experiments were performed at least 3 times. Statistical analysis of data was performed using GraphPad Prism 7 Software (GraphPad Software, Inc., San Diego CA). Data were represented as mean ± standard error of the mean (SEM). An unpaired t-test was performed for comparing the difference between two groups. Before analysing unpaired t-test, F test was used to compare variances. If significant, Welch's test was used to verify. One-way analysis of variance (ANOVA) was used for multiple groups and, if significant, multiple comparisons were further conducted. A *p*-value of <0.05 was considered statistically significant.

## Results

### T1DM Influences General Phenotypes and Corneal Re-Epithelialization

**FIGURE 1:**
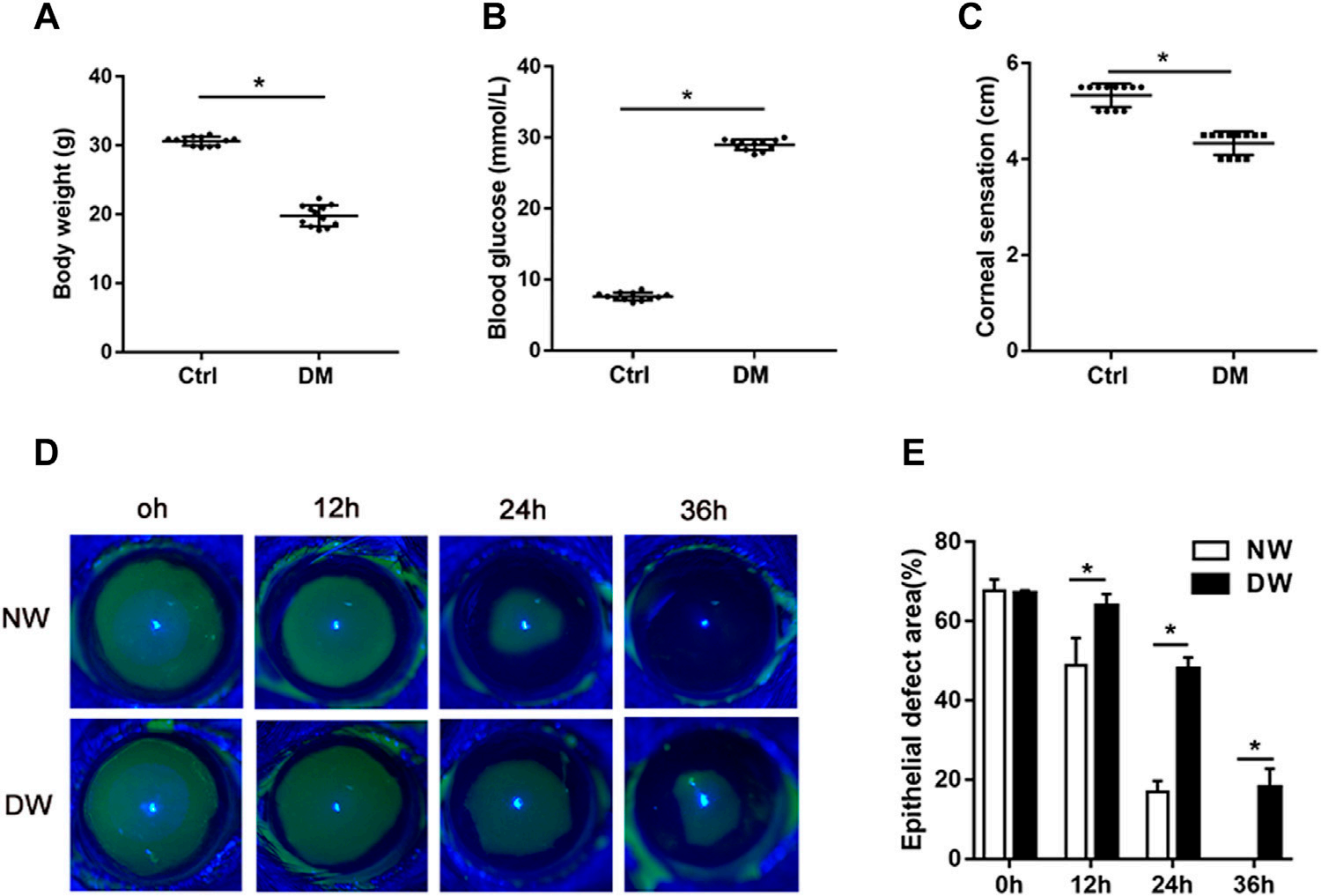
T1DM animal model and general phenotypes. **(A)** The body weight, **(B)** blood glucose levels, and **(C)** corneal sensation levels of normal (Ctrl) and diabetic (DM) mice. **(D)** Fluorescence staining assay was used to record the wound healing process of normal mice with corneal epithelial injury (NW group) and diabetic mice with corneal epithelial injury (DW group). **(E)** The epithelial defect areas were calculated by Image J software. Data were analyzed by unpaired t-test. * *p*-value of <0.05.

Consistent with our previous observation, STZ-treated mice had significant diabetic corneal neuropathy and delayed corneal epithelial regeneration (Bu et al., 2019; Li et al., 2020b; Zhang et al., 2020a). After 16 weeks of STZ injection, we recorded the body weight and blood glucose levels of T1DM and control mice. Expectedly, diabetic mice had significantly less body weight and higher concentrations of blood glucose than those of age-matched control mice (Figures 1A,B). Measurement of corneal sensitivity is commonly used for evaluating corneal nerve function (Yang et al., 2014; Wang et al., 2020a) and the results indicated that diabetic mice had lower corneal sensitivity (Figure 1C). Fluorescein staining further showed that the corneal epithelial healing rate exhibited a significant difference at 12, 24, and 36 h after corneal epithelium scrape. Notably, diabetic mice presented clearly delayed corneal epithelial regeneration (Figures 1D,E). Taken together, our results confirmed that STZ-treated mice possessed characteristics of DK similar to those of diabetic patients. Thus, we obtained samples from T1DM mouse model with corneal epithelial debridement to perform subsequent RNA sequencing.

### RNA Sequencing Identified DEmiRNAs and DEmRNAs in Repaired Corneal Epithelium

**FIGURE 2:**
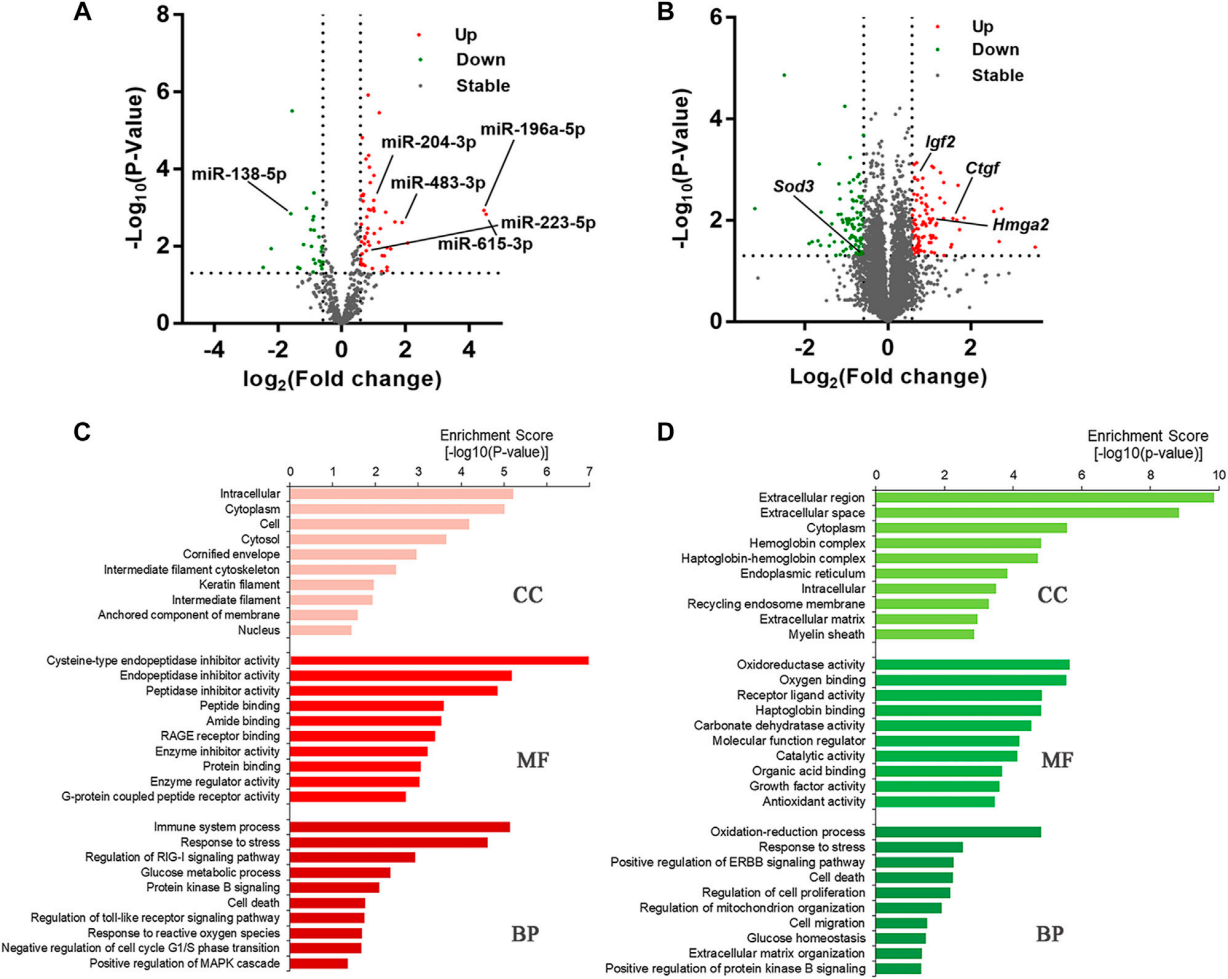
T1DM alters the transcriptome composition of regenerative corneal epithelium. **(A)** DEmiRNAs and **(B)** DEmRNAs are illustrated by volcano plots. **(C)** Gene Ontology (GO) enrichment analysis revealed the cellular component (CC), biological processes (BP) and molecular functions (MF) in which upregulated DEmRNAs and **(D)** downregulated DEmRNAs are significantly enriched.

In order to investigate the potential miRNAs and mRNAs involved in delayed diabetic corneal epithelial regeneration, we conducted the next-generation sequencing (NGS) on corneal epithelium from control and STZ-treated mice 24 h after epithelial debridement. Based on the above cut-off criteria, a total of 77 DEmiRNAs (Supplementary Table S1) and 186 DEmRNAs (Supplementary Table S2) were screened (Figures 2A,B). Some differentially expressed genes were related to complications caused by diabetes. Of the top five up-regulated (miR-615-3p, miR-196a-5p, miR-3095-3p, miR-483-3p, and miR-1946a) and down-regulated (miR-770-3p, miR-138-1-3p, miR-138-5p, miR-1983, and miR-212-3p) miRNAs, some have been described in disorders related to DM. The expression of miR-615 is increased in wounds of diabetic db/db mice and in plasma and skin of patients with DM (Icli et al., 2019). Increased urinary miR-196a might be a prognostic marker of renal fibrosis in patients with diabetic nephropathy (An et al., 2020). An enhanced expression of miR-483-3p in cultured cardiomyocytes under high glucose condition was identified and miR-483-3p may play a pro-apoptotic role (Qiao et al., 2016). Conversely, decreased expression of miR-138 was shown to be associated with obese patients with type 2 diabetes (Pescador et al., 2013). In addition, miR-23b-3p, miR-125b-3p, miR-25-3p, miR-204-5p, miR-10b-5p, and let-7c-5p were also reported to aberrantly expressed in DK (Gao et al., 2015; Kulkarni et al., 2017; Leszczynska et al., 2018). Interestingly, we observed that their clustered and homologous miRNAs (miR-23a-5p, miR-125a-5p, miR-25-5p, miR-204-3p, miR-10a, and let-7days-3p) showed similar alterations in the present study.

Among the DEmRNAs, *Ctgf*, *Hmga2,* and *Igf2* were previously reported to have close interactions with disorders caused by chronic hyperglycemia (Sireesha et al., 2009; Zhu et al., 2019b; Huang et al., 2020). As a member of the antioxidant defense system, *SOD3* has been noted to be downregulated in the serum and urine of patients with diabetes (Kuo et al., 2019). Moreover, we also identified some new genes associated with DK, such as *Fkbp5*, *Fbxo,* and *Cdkn2a*, which could provide new targets for further research. Functional enrichment analysis was carried out to make a thorough investigation for the function of the DEmRNAs (Figures 2C,D). At the BP level, DEmRNAs were identified that participate in regulating immune system processes, the toll-like receptor signaling pathway, MAPK cascade, oxidation-reduction process, cell proliferation, and cell death processes. These results indicated that the DEmRNAs were closely linked with diabetes-related biological processes.

### Potential miRNA/mRNA Pairs Were Selected Through Target Prediction and qRT-PCR

**FIGURE 3:**
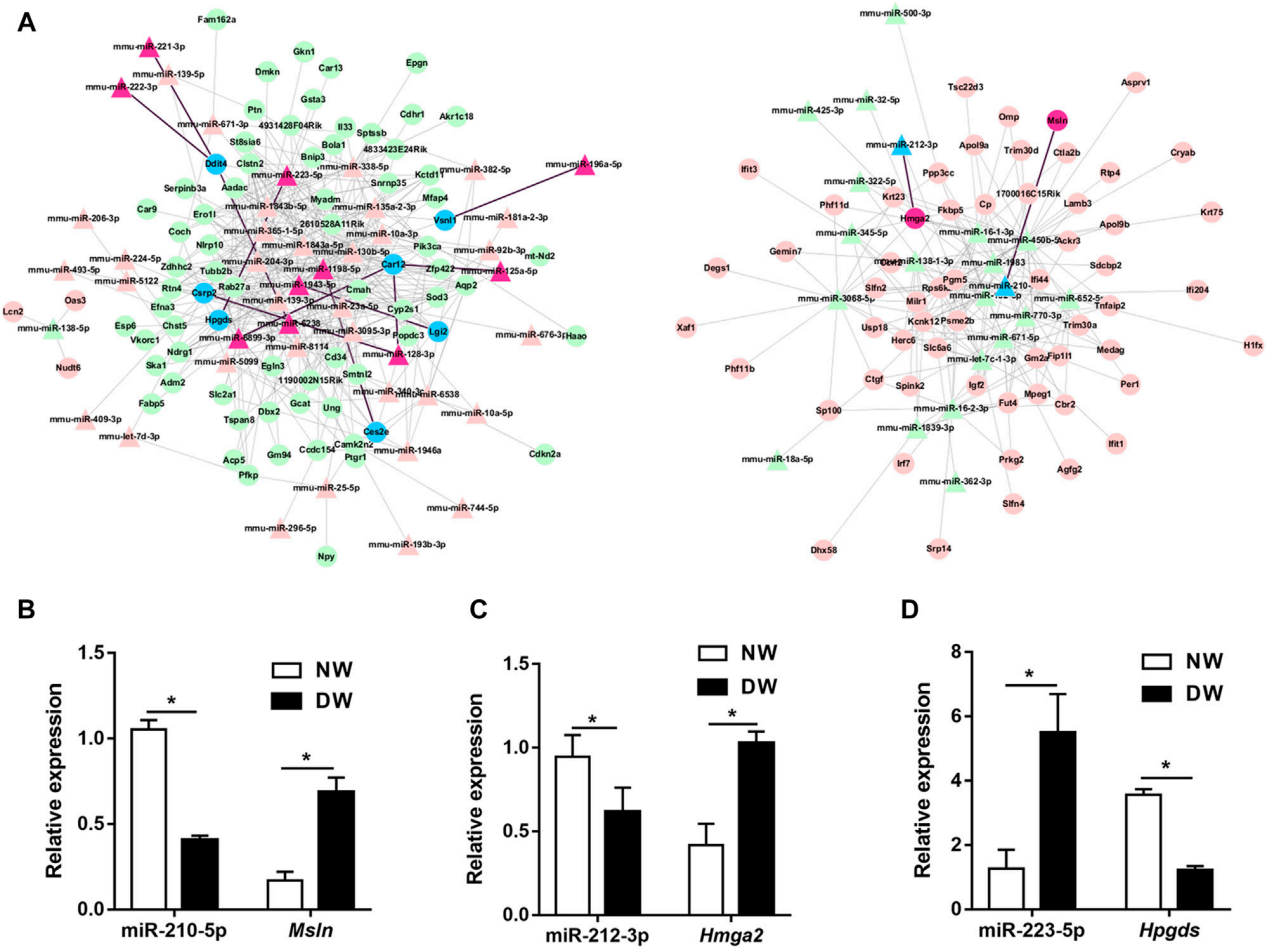
miRNA/mRNA regulatory network analysis. **(A)** Interaction of the miRNA and mRNA were predicted by TargetScan. The upregulated genes are indicated in pink and red, while the downregulated genes are in green and blue. The relatively credible axes are filtered by TargetScan and miRDB, and further selected by scores. Red regions, blue regions and black lines represented the filtered miRNA/mRNA pathways. **(B–D)** miR-210-5p/*Msln*, miR-212-3p/*Hmga2,* and miR-223-5p/*Hpgds* axes were preliminarily validated by qRT-PCR. Data were analyzed by unpaired t-test. * *p*-value of <0.05.

To further investigate the regulatory functions of miRNAs during delayed diabetic corneal re-epithelialization. We used queried established databases to screen miRNA/mRNA pairs. TargetScan is a common software which was widely used for predicting the biological targets of miRNAs. As an initial step, miRNA/mRNA regulatory networks were constructed by means of TargetScan, which included 555 miRNA-mRNA regulatory pairs. Of these, 65 miRNAs targeted 135 mRNAs (Figure 3A). Furthermore, to reduce the probability of false positive results, we combined TargetScan and miRDB to conduct target prediction and selected credible target genes by scores. The results identified nine mRNAs and 12 miRNAs, resulting in 13 miRNA/mRNA interacting pairs (connecting through black straight lines in Figure 3A). These miRNA/mRNA pairs are also emphasized in Table 3.

**TABLE 3:** miRNA/mRNA axes screened out by bioinformatics analysis.

miRNA	Regulation	Target gene	Regulation
miR-1198-5p	Up	Ces2e	down
miR-125a-5p	Up	Car12	down
miR-128-3p	Up	Car12	down
miR-128-3p	Up	Csrp2	down
miR-1943-5p	Up	Lgi2	down
miR-196a-5p	Up	Vsnl1	down
miR-210-5p	down	Msln	up
miR-212-3p	down	Hmga2	up
miR-221-3p	Up	Ddit4	down
miR-222-3p	Up	Ddit4	down
miR-223-5p	up	Hpgds	down
miR-6238	up	Ddit4	down
miR-6899-3p	up	Car12	down

We then used qRT-PCR for preliminary validation of the expression of the 13 miRNA/mRNA pairs. In accordance with our sequencing data, miRNA-125a-5p, miRNA-128-3p, miRNA-221-3p, miRNA-222-3p, miRNA-223-5p, *Msln*, and *Hmga2* significantly increased while miRNA-210-5p, miRNA-212-3p, *Ces2e*, *Lgi2*, *Vsnl1*, and *Hpgds* were found to be downregulated in the regenerative T1DM corneal epithelium. Notably, although the expression differences of miRNA-1943-5p, miRNA-196a-5p, miRNA-6238, and *Csrp2*, between diabetic and normal mice did not reach statistical significance, they demonstrated comparable expression trends in sequencing and qRT–PCR analyses. In addition, *Ddit4* increased in diabetic group, implying a divergent pattern of *Ddit4* between sequencing and validation. Thus, based on the results of our multistep analysis and qRT-PCR, miR-210-5p/*Msln*, miR-212-3p/*Hmga2,* and miR-223-5p/*Hpgds* pairs were implicated in the pathogenesis of delayed diabetic corneal wound closure (Figures 3B–D).

### Suppressing miR-223-5p Promoted Diabetic Delayed Corneal Epithelial and Nerve Regeneration

**FIGURE 4:**
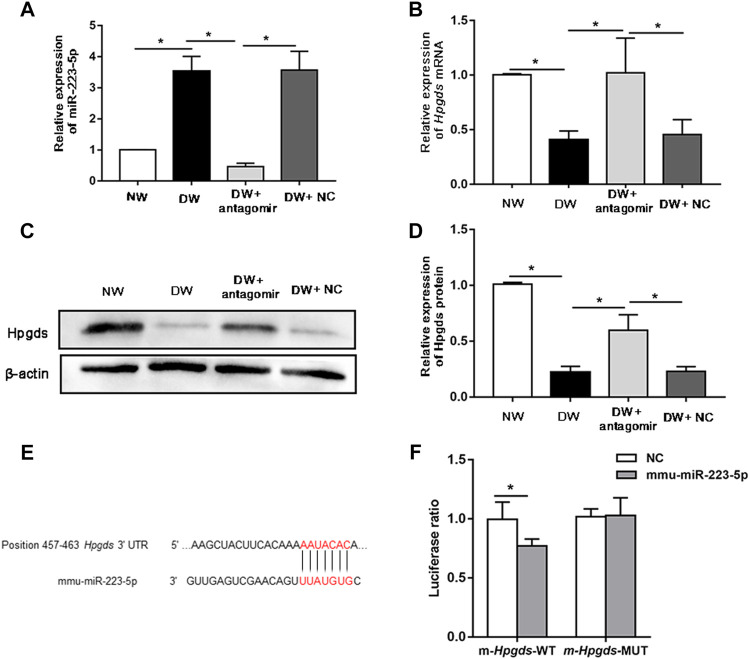
*Hpgds* is the direct target gene of miR-223-5p. **(A)** qRT-PCR results revealing miR-223-5p expression of in the corneal epithelium harvested 24 h post-wounding of normal mice (NW group), diabetic mice (DW group), diabetic mice subconjunctivally injected with miR-223-5p antagomir (DW + antagomir group) or antagomir NC (DW + NC group). **(B)** qRT-PCR results revealing mRNA expression of *Hpgds* in the corneal epithelium 24 h post-wounding of the same four groups. **(C,D)** Western blotting showing the protein expression of *Hpgds* in the cornea 24 h post-injury of these four groups. **(E)** Schematic representation of the predicted interacting site of mmu-miR-223-5p with *Hpgds*. **(F)** Luciferase reporter assay assessed the relative luciferase activity in HEK293T cells transfected with pMIR-REPORT *Hpgds*-WT-3′-UTR or *Hpgds*-Mut-3′-UTR upon transfection with miR-223-5p mimic or miRNA mimic NC. **(A,B,D)** Data were analyzed by one-way ANOVA. If significant, multiple comparations were then performed. **(F)** Data were analyzed by unpaired t-test. *p-value of <0.05.

Of these miRNA/mRNA pairs mentioned above, miR-223-5p/*Hpgds* attracted our attention due to the most significant shift of miR-223-5p. Moreover, miR-223 was reported to play a role in disorders induced by DM (Deng et al., 2020; Xu D. et al., 2020) and *Hpgds* can increase insulin sensitivity (Fujitani et al., 2010). However, little is known about the detailed regulatory function of miR-223-5p/*Hpgds* in DK. Herein, we firstly attempted to validate the association between miR-223-5p and *Hpgds*. Subconjunctival injection of miR-223-5p antagomir was utilized to block miR-223-5p in the cornea of mice. As expected, miR-223-5p antagomir successfully suppressed the upregulation of miR-223-5p in diabetic regenerated corneal epithelium (Figure 4A). qRT-PCR also indicated that inhibiting miR-223-5p enhanced the mRNA levels of *Hpgds* (Figure 4B). Similar to the observations on PCR assays, miR-223-5p antagomir treatment stimulated the Hpgds proteins in diabetic wounded cornea (Figures 4C,D). A luciferase reporter assay indicated that miR-223-5p inhibited luciferase expression of the transcript including the wild-type 3′-UTR of *Hpgds* but not that of the transcript including the seed sequence-mutated 3′-UTR (Figures 4E,F). These results revealed that, in line with the aforementioned analysis, miR-223-5p can directly inhibit the expression of *Hpgds*.

**FIGURE 5:**
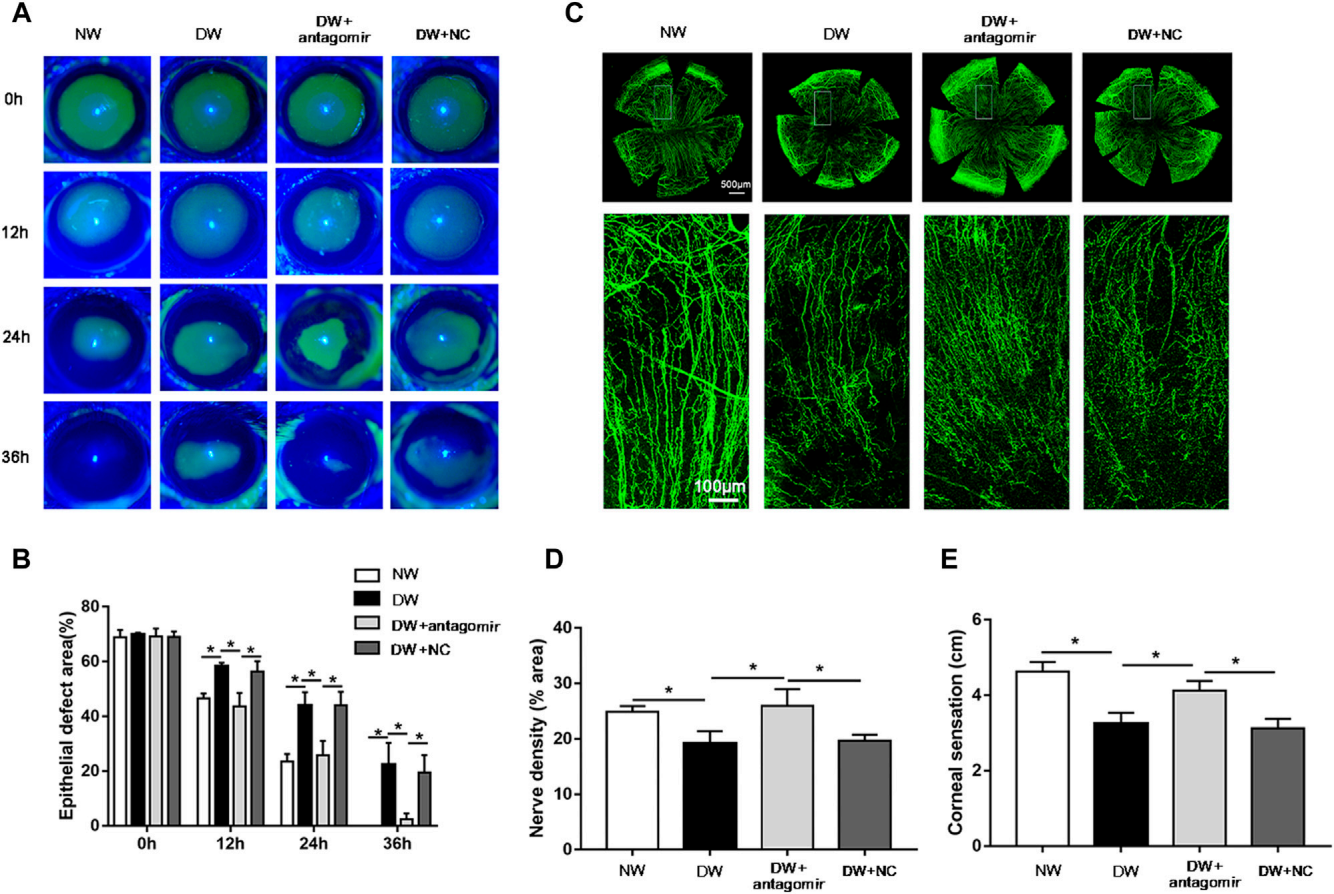
Inhibition of miR-223-5p ameliorated corneal epithelial wound repair and nerve regeneration in diabetic mice. **(A,B)** The fluorescence staining assay exhibited the corneal epithelial defect of normal mice (NW group), diabetic mice (DW group), diabetic mice subconjunctivally injected with miR-223-5p antagomir (DW + antagomir group), or antagomir NC (DW + NC group) at 0, 12, 24, and 36 h post-injury. **(C,D)** Corneal whole-mount staining of β-tubulin III revealing the corneal nerves of the above four groups on day 5 post-wounding. **(E)** The corneal sensation measurement was performed on day 5 post-wounding in these four groups. **(B,D,E)** Data were analyzed by one-way ANOVA. If significant, multiple comparations were then performed. *p-value of <0.05.

Considering the high expression of miR-223-5p in diabetic regenerative corneal epithelium, it is reasonable for us to detect the potential role of miR-223-5p during re-epithelialization. First, we found that the defect size of the corneal epithelium in the miR-223-5p antagomir-treated diabetic mice was obviously reduced compared with that of the diabetic mice and the miRNA antagomir NC-treated diabetic mice at 12, 24, and 36 h after injury, which nearly reached a comparable level to that of the control mice (Figures 5A,B). Since the terminal endings of corneal nerves are distributed in the corneal epithelium and they mutually maintain the growth of each other (Di et al., 2017), we next detected the impact of miR-223-5p on corneal nerve regeneration. On day 5 after corneal epithelial injury, the diabetic mice that had received miR-223-5p antagomir subconjunctival injection showed higher corneal nerve density than the diabetic mice and miRNA antagomir NC-treated diabetic mice (Figures 5C,D). Moreover, corneal sensation detection is a method used to evaluate corneal nerve function. In parallel with the results of corneal whole-mount staining, we observed an increase in corneal sensation in diabetic mice treated by miR-223-5p antagomir (Figure 5E). Taken together, miR-223-5p might serve as an important regulator during diabetic corneal epithelial and nerve regeneration.

### Inhibition of Hpgds Counteracted the Protective Effects of miR-223-5p Antagomir

**FIGURE 6:**
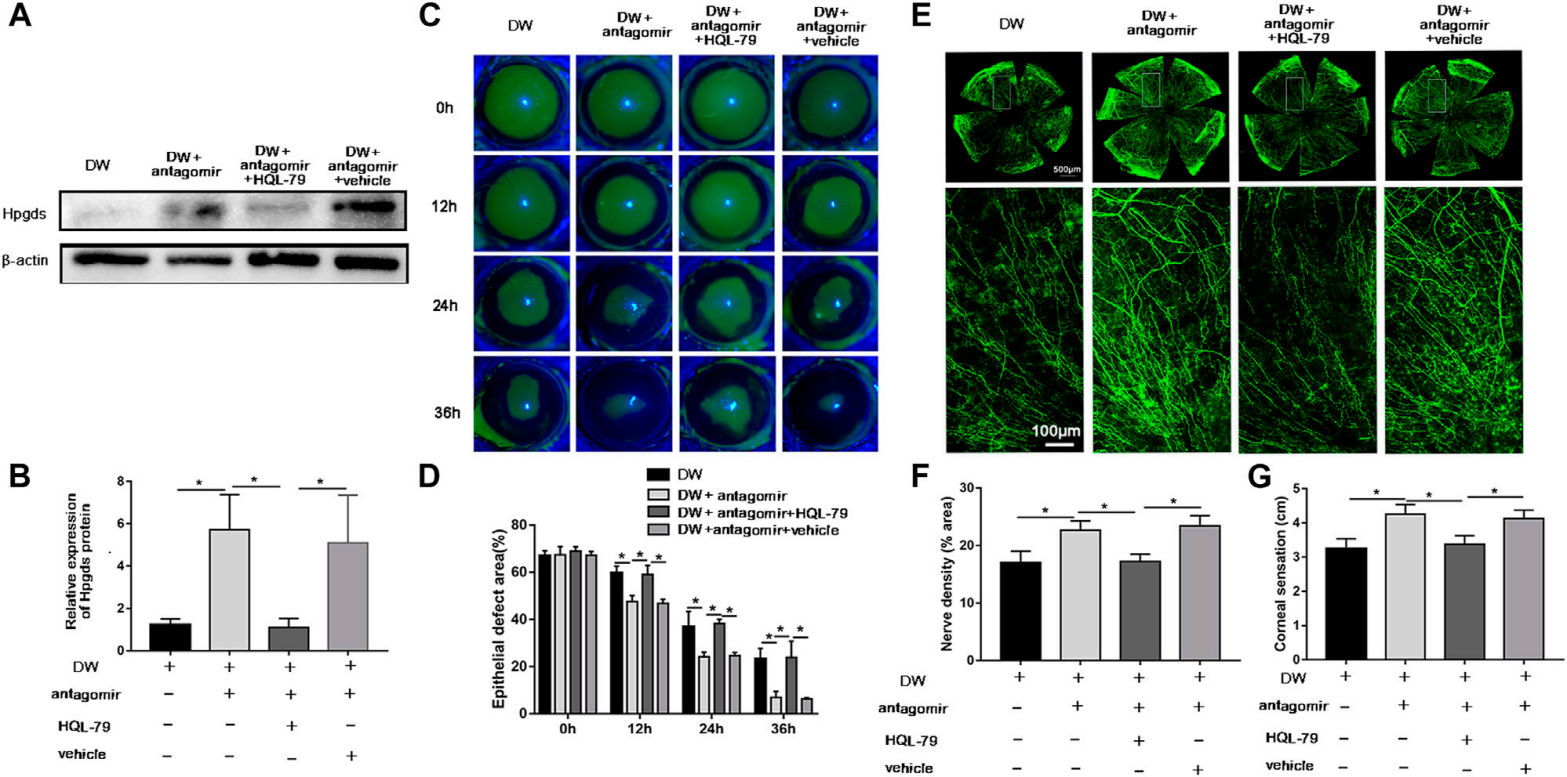
Blockade of Hpgds counteracts the beneficial effects induced by miR-223-5p antagomir. **(A,B)** Western blotting assay showing the protein expression of Hpgds in the cornea 24 h post-wounding of diabetic mice (DW group), miR-223-5p antagomir treated diabetic mice (DW + antagomir group), miR-223-5p antagomir and HQL-79 treated diabetic mice (DW + antagomir + HQL-79 group), miR-223-5p antagomir, and vehicle treated diabetic mice (DW + antagomir + vehicle group). **(C,D)** Fluorescence staining assay showing the corneal epithelial defect areas of DW group, DW + antagomir group, DW + antagomir + HQL-79 group and DW + antagomir + vehicle group at different timepoints post-injury. **(E,F)** Corneal whole-mount staining of β-tubulin III revealing the corneal nerves of each group on day 5 post-wounding. **(G)** Corneal sensation measurement was conducted on day 5 post-wounding in each group. **(B,D,F,G)** Data were analyzed by One-way ANOVA. If significant, multiple comparations were then performed. * *p*-value of <0.05.

Since *Hpgds* was identified as a downstream target of miR-223-5p, we next sought to determine whether inhibition of *Hpgds* could disrupt the effects of miR-223-5p antagomir on diabetic corneal wound healing. HQL-79, a specific Hpgds inhibitor (Redensek et al., 2011), markedly antagonized the promotion effects of miR-223-5p antagomir on Hpgds protein expression, suggesting HQL-79 could successfully downregulate the expression of Hpgds in diabetic wounded corneas (Figures 6A,B). Furthermore, we determined that the corneal injured area of diabetic mice treated by miR-223-5p antagomir and HQL-79 was larger at 12, 24, and 36 h after wounding, compared with injured areas of diabetic mice receiving miR-223-5p antagomir with or without vehicle treatment (Figures 6C,D). Consistently, 5 days after corneal epithelial injury, HQL-79 could also reverse the effects of miR-223-5p antagomir on promoting diabetic corneal nerve regeneration and diabetic corneal sensitivity recovery (Figures 6E–G). Therefore, these findings demonstrated that miR-223-5p plays a role in diabetic corneal epithelial and nerve regeneration mediating by *Hpgds*.

## Sequestration of miR-223-5p Attenuated Inflammatory Response and Promoted Proliferation Signals During Diabetic Corneal Wound Healing

**FIGURE 7:**
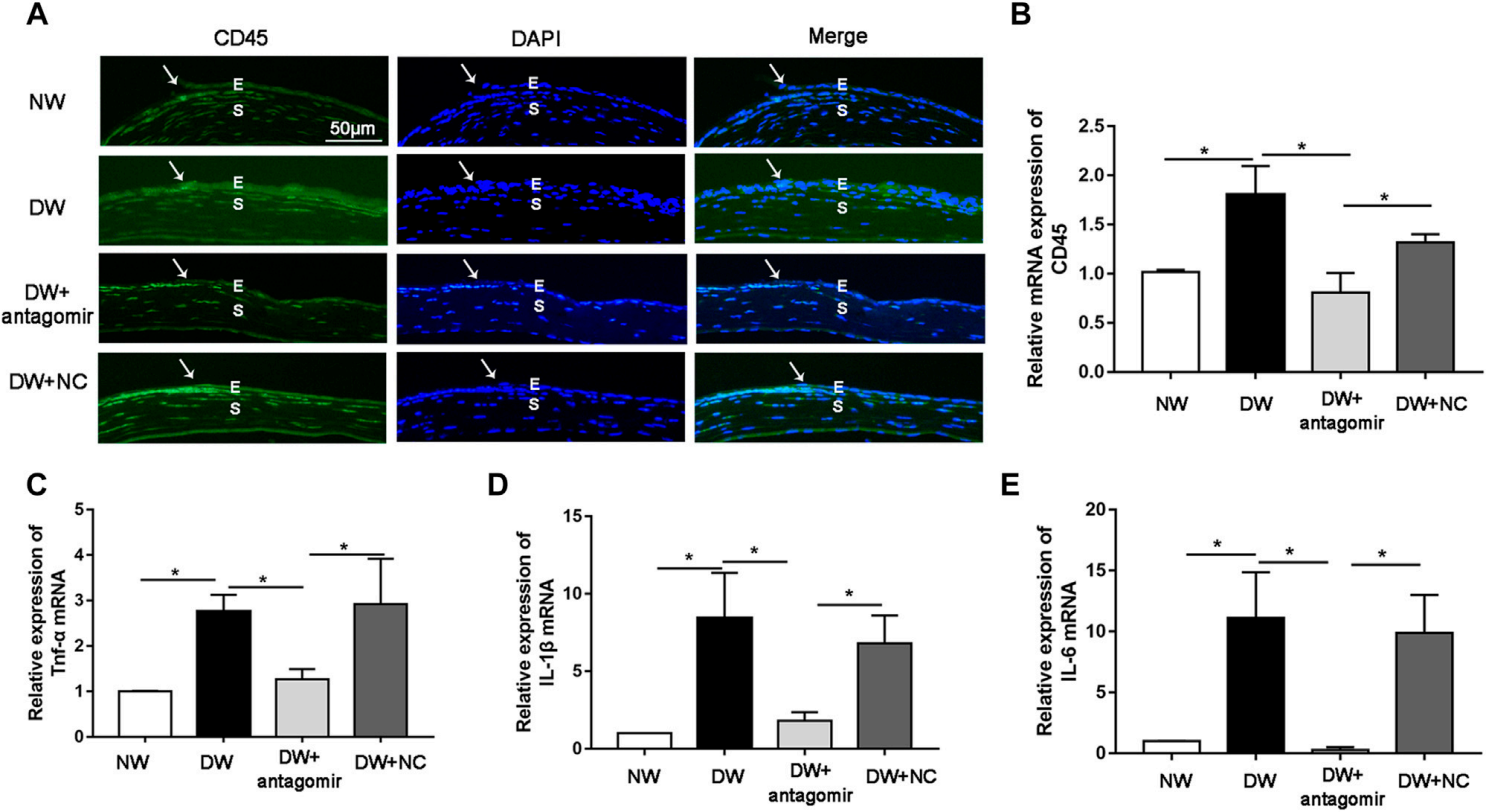
miR-223-5p antagonist promoted diabetic corneal epithelial wound repair and nerve regeneration by modulating inflammatory process. **(A)** Immunofluorescence corneal staining reflected CD45 protein expression in the normal mice (NW group), diabetic mice (DW group), diabetic mice subconjunctivally injected with miR-223-5p antagomir (DW + antagomir group) or antagomir NC (DW + NC group) 24 h post-injury. **(B)** qRT-PCR results revealing the CD45 mRNA expression of the corneal epithelium in each group. **(C–E)** qRT-PCR detected the mRNA expression of inflammatory factors including TNF-α, IL-1β, and IL-6 in the corneal epithelium of these four groups at 24 h post-wounding. **(B–E)** Data were analyzed by one-way ANOVA. If significant, multiple comparations were then performed. * *p*-value of <0.05.

The data described above revealed a role for miR-223-5p during corneal re-epithelialization; therefore, we sought to determine the mechanism by which miR-223-5p disturbed tissue regeneration. Recent studies have reported that inflammatory cytokines including IL-1β and TNF-α caused defective corneal epithelial repair in diabetic mice (Wang et al., 2020b), indicating that excessive inflammatory response is a major inducing factor for delayed wound healing. Since miR-223 plays an essential role in inflammation (Yuan et al., 2018), we assumed that a miR-223-5p antagomir could have an impact on inflammatory processes in DK pathogenesis. Immunofluorescence results revealed that the staining intensity of CD45 (pan-leukocyte marker) was stronger in diabetic corneas compared with control corneas 24 h after wounding, while miR-223-5p antagomir administration significantly reduced the staining intensity in diabetic mice (Figure 7A). Consistently, qRT-PCR indicated that CD45 mRNA levels was increased in diabetic corneal epithelium collected 24 h after wounding compared with control group, whereas, miR-223-5p antagomir decreased the CD45 mRNA levels in diabetic wounded corneal epithelium (Figure 7B). In addition, the results of qRT-PCR showed that suppression of miR-223-5p downregulated overexpressed inflammatory factors, including IL-1β, IL-6, and TNF-α (Figures 7C–E). In summary, our findings suggested the inflammation-suppressive effect of miR-223-5p blockade on diabetic corneal wound healing.

**FIGURE 8:**
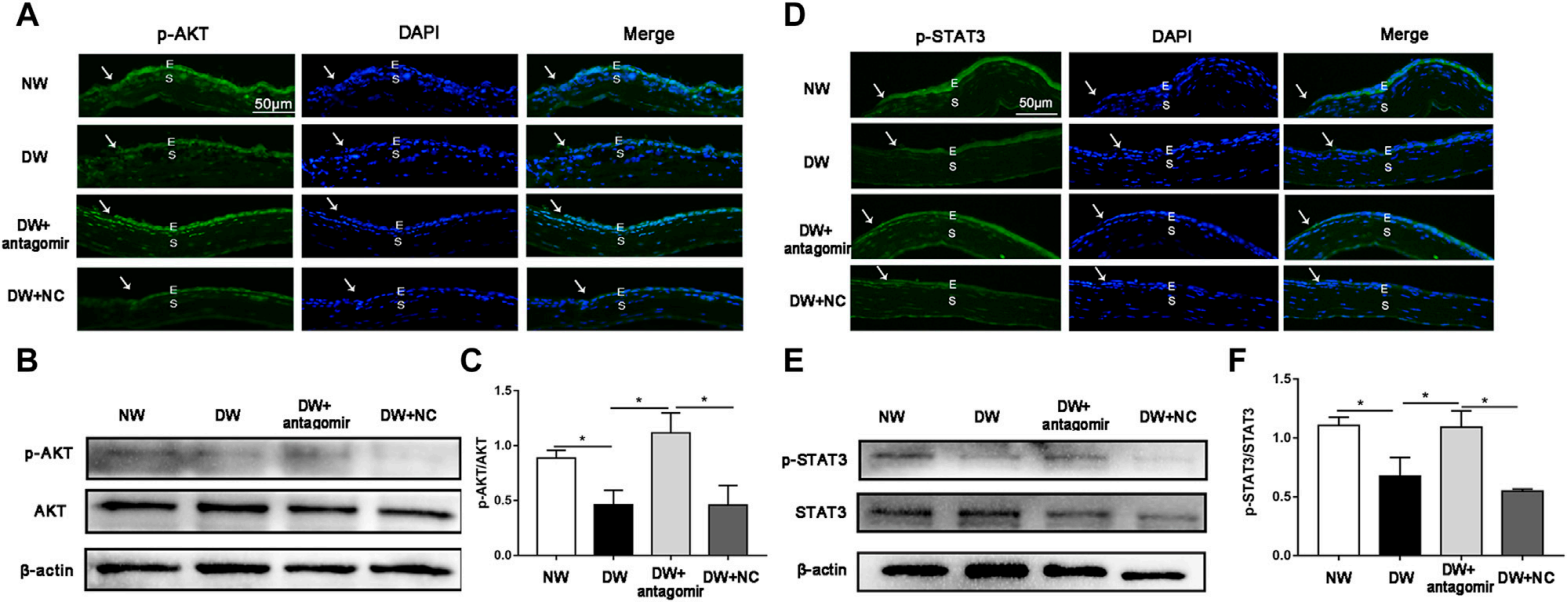
miR-223-5p antagomir improved the phosphorylation of AKT and STAT3. **(A)** Immunofluorescence corneal staining showing the p-AKT protein expression in normal mice (NW group), diabetic mice (DW group), and diabetic mice subconjunctivally injected with miR-223-5p antagomir (DW + antagomir group) or antagomir NC (DW + NC group) 24 h post-injury. **(B,C)** Western blotting assay measured the p-AKT protein levels of the cornea 24 h post-injury in each group. **(D)** Immunofluorescence corneal staining and **(E,F)** western blotting assay showing p-STAT3 protein expression in the same four groups 24 h post-wounding. **(C,F)** Data were analyzed by one-way ANOVA. If significant, multiple comparations were then performed. * *p*-value of <0.05.

Moreover, phosphorylation of AKT (p-AKT) and STAT3 (p-STAT3) which are classical proliferative signaling-related molecules, have been reported to significantly decrease during diabetic corneal wound healing (Li et al., 2020a; Li et al., 2020b; Wang et al., 2020b). Thus, we investigated whether inhibiting miR-223-5p could influence the expression of p-AKT and p-STAT3. Immunofluorescence and western blotting demonstrated that depletion of miR-223-5p promoted the recovery of p-AKT and p-STAT3 in diabetic wounded corneas, suggesting that cell proliferation is also a mechanism mediated by miR-223-5p in DK (Figure 8).

## Discussion

DM is a common systemic disease with rising prevalence (Markoulli et al., 2018). Disorders caused by DM are a serious public health challenge and financial burden to many countries (Mansoor et al., 2020). As a complication induced by DM, DK has gained increasing attention recently, and can lead to delayed corneal epithelium and nerve regeneration (Li et al., 2020a; Wang et al., 2020a; Li et al., 2020b). The molecular mechanism involved in DK is intricate, highlighting the importance of constructing the intermolecular regulatory networks regulating its pathobiology. Studies that have sequenced mRNAs and miRNAs simultaneously are uncommon, even though their strength in identifying miRNA targets is well acknowledged. In our investigation, the corneal epithelial debridement model was established in normal and diabetic mice models. Regenerated epithelium was collected 24-h post-injury and the expression of miRNA/mRNA pairs involved in the process of diabetic corneal epithelial regeneration were detected by simultaneously performing miRNA and mRNA sequencing, and the miR-223-5p/*Hpgds* pair was selected with subsequent mechanistic studies.

Starting from NGS, we obtained 77 DEmiRNAs and 186 DEmRNAs responsible for delayed wound healing of diabetic corneal epithelium. Following a literature research, DEmiRNAs and DEmRNAs reported to have a close relationship with hyperglycemia-related disorders were identified. Our study confirmed their involvement in DK. In addition, we also identified some DEmiRNAs and DEmRNAs that had not been previously reported in complications linked to DM, which could offer new targets for future investigations. To gain insight into the molecular underpinnings of these DEmRNAs, we performed functional analyses. The GO analysis revealed that several significant BP terms were closely related to DM, including “glucose metabolic process” and “glucose homeostasis.” We also identified a number of BP terms associated with cell proliferation and immune reactions, such as the “cell death,” “protein kinase B signaling,” “negative regulation of cell cycle G1/S phase transition,” “regulation of toll-like receptor signaling pathway” and “immune system process.”

Having both miRNA sequencing and mRNA sequencing data from the same sample offers a strong advantage to explore credible and meaningful miRNA/mRNA pairs. By means of a multistep analysis and qRT-PCR assay, the miR-210-5p/*Msln*, miR-212-3p/*Hmga2,* and miR-223-5p/*Hpgds* pairs were preliminarily validated with relatively high reliability. Of these three axes, miR-223-5p was the miRNA with the largest fold change between control mice and diabetic mice. In addition, miR-223 is well known as an essential cell regulator and has been implicated in mediating infection, inflammation, different cancer types, and diabetes (Haneklaus et al., 2013; Sánchez-Ceinos et al., 2021). *Hpgds* also participates in regulating insulin sensitivity and inflammatory diseases (Fujitani et al., 2010; Huang et al., 2021). Thus, we focused on miR-223-5p/*Hpgds* pathway for further mechanistic investigation.

The miR-223-5p/*Hpgds* axis has not been described previously. We provided evidenced that inhibition of miR-223-5p could upregulate the expression of *Hpgds*, and *Hpgds* is directly targeted by miR-223-5p. Accumulating evidence has implied that miR-223 plays a role in DM-related disorders. AGE accumulation, a characteristic pathological change of DM, was reported to promote apoptosis with up-regulation of miR-223 (Qin et al., 2016). miR-223 may represent a therapeutic target in AGE-induced injury to osteoblasts in DM (Qin et al., 2016). Shao et al. revealed that the expression of miR-223-3p was higher in the serum and aqueous humor of DR patients as compared with that of nondiabetic subjects and miR-223-3p levels were consistent with the severity of DR, indicating that miR-223-3p could serve as a potential biomarker in the progression of DR (Shao et al., 2019). More recently, miR-223 was found to exert regulatory effects on diabetic cardiomyopathy (Deng et al., 2020; Xu D. et al., 2020). The role of miR-223-5p in DK has not been explored to date. Herein, we observed that miR-223-5p antagomir treatment promoted diabetic corneal epithelial and nerve regeneration, and this positive effect can be reversed by inhibiting Hpgds, which points towards a potential therapeutic value to DK.

MiRNAs are known to target hundreds of genes to regulate multiple biological process (Selbach et al., 2008). We continued to explore the potential pathways mediated by miR-223-5p during diabetic corneal re-epithelialization. Inflammatory response is a common phenomenon during the process of corneal regeneration. By screening for the intersection of miR-223-5p target genes and inflammatory response gene sets, we performed interaction network analysis and found that miR-233-5p did regulate a series of inflammatory genes (data not shown). In our future work, we will continue to explore and validate the detailed regulation mechanism. On the other hand, in spinal cord injury, miR-223-5p inhibitor significantly suppressed the expressions of inflammatory factors, including IL-1β, IL-6, and TNF-α (Guan et al., 2019). Herein, we observed that suppressing miR-223-5p can relieve inflammation in diabetic corneal wound healing, hinting that miR-223-5p might play a pro-inflammatory role in DK.

Previous observations have documented that levels of p-AKT and p-STAT3 were decreased in diabetic corneas compared with levels found in control group (Xu et al., 2009; Li et al., 2020a; Li et al., 2020b), and the influence of miR-223 on p-AKT and p-STAT3 have also been reported. For example, the expression of miR-223 was significantly enhanced in the livers of high-fat diet-fed mice and overexpressed miR-223 can decrease the level of p-AKT (Zhang et al., 2020b). In experimental colitis and Kawasaki disease, overexpression of miR-223 can decrease the levels of p-STAT3 (Wang et al., 2019; Zhang et al., 2021). Furthermore, our study found that an miR-223-5p antagomir could restore the reduce the expression of p-AKT and p-STAT3 in diabetic corneas.

In conclusion, our findings unveiled that miR-223-5p is a regulatory gene involved in diabetic corneal epithelial and nerve regeneration and mediates inflammatory processes and cell proliferation signaling. Considering that miRNAs have significant therapeutic potential, targeting miR-223-5p individually or combination with other pivotal miRNAs discovered previously may contribute to the development of therapeutic strategies for improving DK. It should be noted that the specific role of Hpgds in DK should be explored in the near future, and the other miRNA/mRNA pairs identified in our study also need further investigations. Generally speaking, this study provides important insight for understanding DK pathogenesis, and may provide potential diagnostic biomarkers or therapeutic targets for DK interventions.

## Data Availability

The datasets presented in this study can be found in online repositories. The names of the repository/repositories and accession number(s) can be found below: https://www.ncbi.nlm.nih.gov/geo/query/acc.cgi?acc=GSE180634.
