# Supplementary material for: Transcriptional Network Analysis Reveals the Role of miR-223-5p During Diabetic Corneal Epithelial Regeneration
Source: Front Mol Biosci. 2021 Aug 26;8:737472. doi: 10.3389/fmolb.2021.737472 (PMC8427436; doi:10.3389/fmolb.2021.737472)
Supplement: Supplementary file 1 [file Table1.docx]

Supplementary Table 1 DEmiRNAs between normal and diabetic [regenerative](javascript:;) corneal epithelium

| Upregulated DEmiRNA | Log2FC | Downregulated DEmiRNA | Log2FC |
| --- | --- | --- | --- |
| mmu-miR-615-3p | 4.531816676 | miR-770-3p | -2.460255482 |
| mmu-miR-196a-5p | 4.464638569 | mmu-miR-138-1-3p | -2.211201254 |
| mmu-miR-3095-3p | 2.072542371 | mmu-miR-138-5p | -1.59745496 |
| mmu-miR-483-3p | 1.892959034 | mmu-miR-1983 | -1.555014988 |
| mmu-miR-1946a | 1.674752448 | mmu-miR-212-3p | -1.371230882 |
| mmu-miR-6238 | 1.540944992 | mmu-miR-652-5p | -1.31028196 |
| mmu-miR-6899-3p | 1.423730311 | mmu-miR-451a | -1.197656646 |
| mmu-miR-6538 | 1.422736102 | mmu-miR-450b-5p | -1.107912457 |
| mmu-miR-5122 | 1.418334019 | mmu-miR-1839-3p | -0.967968464 |
| mmu-miR-365-1-5p | 1.383148802 | mmu-miR-671-5p | -0.944860798 |
| mmu-miR-139-3p | 1.335219009 | mmu-miR-3068-5p | -0.907227712 |
| mmu-miR-10a-3p | 1.267887782 | mmu-miR-500-3p | -0.897999144 |
| mmu-miR-493-5p | 1.253410387 | mmu-miR-210-5p | -0.87878265 |
| mmu-miR-23a-5p | 1.21161721 | mmu-miR-3102-3p | -0.875320688 |
| mmu-miR-222-3p | 1.176668147 | mmu-miR-322-5p | -0.867288917 |
| mmu-miR-135a-2-3p | 1.161998463 | mmu-miR-345-5p | -0.858508201 |
| mmu-miR-223-3p | 1.018741108 | mmu-miR-16-2-3p | -0.82640722 |
| mmu-miR-224-5p | 1.015969473 | mmu-miR-425-3p | -0.71528303 |
| mmu-miR-1843b-3p | 1.009573101 | mmu-let-7c-1-3p | -0.700092416 |
| mmu-miR-204-3p | 1.000138559 | mmu-miR-18a-5p | -0.660019626 |
| mmu-miR-10a-5p | 0.992416596 | mmu-miR-362-3p | -0.644882081 |
| mmu-miR-1943-5p | 0.965112747 | mmu-miR-450a-5p | -0.640141107 |
| mmu-miR-128-3p | 0.8934552 | mmu-miR-152-5p | -0.622953042 |
| mmu-miR-25-5p | 0.891793447 | mmu-miR-16-1-3p | -0.618328376 |
| mmu-miR-676-3p | 0.874244971 | mmu-miR-32-5p | -0.609353484 |
| mmu-miR-223-5p | 0.872653526 | mmu-miR-34a-5p | -0.591244246 |
| mmu-miR-181a-2-3p | 0.848637584 |  |  |
| mmu-miR-5099 | 0.842117273 |  |  |
| mmu-miR-125a-5p | 0.832092183 |  |  |
| mmu-miR-139-5p | 0.826778744 |  |  |
| mmu-miR-92b-3p | 0.823318771 |  |  |
| mmu-miR-151-5p | 0.794623377 |  |  |
| mmu-miR-193b-3p | 0.78926506 |  |  |
| mmu-miR-130b-5p | 0.767208027 |  |  |
| mmu-miR-744-5p | 0.76485388 |  |  |
| mmu-miR-296-3p | 0.729742433 |  |  |
| mmu-miR-671-3p | 0.726485428 |  |  |
| mmu-miR-296-5p | 0.725258386 |  |  |
| mmu-miR-382-5p | 0.712168363 |  |  |
| mmu-miR-1843b-5p | 0.682673128 |  |  |
| mmu-miR-338-5p | 0.670600083 |  |  |
| mmu-miR-221-3p | 0.670529615 |  |  |
| mmu-miR-1198-5p | 0.667902786 |  |  |
| mmu-miR-423-3p | 0.644646333 |  |  |
| mmu-miR-206-3p | 0.635998502 |  |  |
| mmu-miR-409-3p | 0.628060115 |  |  |
| mmu-miR-340-3p | 0.622114245 |  |  |
| mmu-let-7d-3p | 0.621316941 |  |  |
| mmu-miR-1843a-5p | 0.615321549 |  |  |
| mmu-miR-8114 | 0.603481995 |  |  |
| mmu-miR-1249-3p | 0.593854304 |  |  |
