# Supplementary material for: Transcriptional Network Analysis Reveals the Role of miR-223-5p During Diabetic Corneal Epithelial Regeneration
Source: Front Mol Biosci. 2021 Aug 26;8:737472. doi: 10.3389/fmolb.2021.737472 (PMC8427436; doi:10.3389/fmolb.2021.737472)
Supplement: Supplementary file 2 [file Table2.docx]

Supplementary Table 2 DEmRNAs between normal and diabetic [regenerative](javascript:;) corneal epithelium

| Upregulated DEmiRNA | Log2FC | Downregulated DEmiRNA | Log2FC |
| --- | --- | --- | --- |
| *Gm5483* | 3.556719218 | *Gm21887* | -3.212652714 |
| *Msln* | 2.741604725 | *Bola1* | -2.497657892 |
| *Stfa2l1* | 2.687913751 | *Prl2c2* | -1.899698175 |
| *Stfa2* | 2.555608733 | *Hbb-bs* | -1.827901957 |
| *Slfn4* | 1.843558122 | *Hba-a2* | -1.686343214 |
| *Gm5416* | 1.73049524 | *Zfp422* | -1.654876273 |
| *Ccl5* | 1.69544078 | *Hba-a1* | -1.624045296 |
| *Ifit3* | 1.662136727 | *Gm94* | -1.609813201 |
| *Ctgf* | 1.568352779 | *Wfdc18* | -1.415998429 |
| *Ifit1* | 1.540379369 | *Prl2c3* | -1.38920884 |
| *H1fx* | 1.523100307 | *Sptssb* | -1.353607092 |
| *Ifit3b* | 1.368896832 | *Mmp10* | -1.29166949 |
| *Fpr2* | 1.353168358 | *Defb14* | -1.25222621 |
| *Isg15* | 1.349181307 | *Aadac* | -1.201837183 |
| *Tsc22d3* | 1.343060653 | *Bnip3* | -1.181054015 |
| *Krt23* | 1.26722229 | *Akr1c18* | -1.158368003 |
| *Krt75* | 1.257200702 | *Serpinb2* | -1.152717321 |
| *Slfn1* | 1.172039669 | *4833423E24Rik* | -1.131336452 |
| *Lce3b* | 1.158735218 | *Serpinb3a* | -1.1013125 |
| *Lcn2* | 1.155494323 | *2300002M23Rik* | -1.09797953 |
| *Cryab* | 1.123267091 | *Lgi2* | -1.083746305 |
| *Ifit1bl1* | 1.10182622 | *Il33* | -1.075085496 |
| *Phf11b* | 1.088789226 | *Ndrg1* | -1.040517223 |
| *Hmga2* | 1.083951055 | *Hpgds* | -1.021268739 |
| *Tnfaip2* | 1.077347518 | *Apobec2* | -1.00857507 |
| *Trim30d* | 1.072227505 | *Ankrd37* | -0.999793878 |
| *Trim30a* | 1.06271963 | *Ero1l* | -0.980409914 |
| *Gal* | 1.053169444 | *Cdkn2a* | -0.969420803 |
| *Psg22* | 1.04667961 | *Car12* | -0.953414806 |
| *Apol9a* | 1.039716709 | *Ddit4* | -0.946364644 |
| *Crygn* | 1.037904425 | *Car13* | -0.944324415 |
| *Lce3a* | 1.024880463 | *Slc27a3* | -0.926682115 |
| *Kcnk12* | 1.015027879 | *Mfap4* | -0.913519774 |
| *Usp18* | 0.996118219 | *Aknad1* | -0.909111688 |
| *Apol9b* | 0.994232541 | *Vsnl1* | -0.906084964 |
| *Per1* | 0.947019714 | *Adm2* | -0.900954571 |
| *Rtp4* | 0.941075841 | *Csrp2* | -0.885068991 |
| *1700016C15Rik* | 0.923149592 | *Esp6* | -0.884136242 |
| *Fkbp5* | 0.922279724 | *Cmah* | -0.8834157 |
| *Krt18* | 0.915441887 | *Rtn4* | -0.870865806 |
| *Fut4* | 0.904259734 | *Fabp5* | -0.865853002 |
| *Ddx60* | 0.903095068 | *2610528A11Rik* | -0.853580492 |
| *Slc6a6* | 0.877954952 | *Pfkp* | -0.852877374 |
| *Slfn2* | 0.873087522 | *Lgals7* | -0.845862048 |
| *Ctla2b* | 0.855072433 | *Kctd11* | -0.83374014 |
| *Ifi211* | 0.854656492 | *Egln3* | -0.830655665 |
| *Plk3* | 0.838835195 | *Efna3* | -0.829642038 |
| *Degs1* | 0.829137631 | *Acp5* | -0.82750945 |
| *Zbp1* | 0.828801575 | *Car9* | -0.820562705 |
| *Sp100* | 0.824564816 | *Tspan8* | -0.812397252 |
| *Xaf1* | 0.819097428 | *Haao* | -0.788220284 |
| *Cp* | 0.798024024 | *Fam162a* | -0.777835368 |
| *Omp* | 0.79584582 | *Rab27a* | -0.768997243 |
| *Sdcbp2* | 0.766488568 | *Zdhhc2* | -0.761359165 |
| *Gm42641* | 0.765243888 | *Popdc3* | -0.756155213 |
| *S100a4* | 0.76370566 | *Coch* | -0.741491589 |
| *Spink2* | 0.763354292 | *Cd34* | -0.740494199 |
| *Oas3* | 0.756729061 | *Ces2e* | -0.73924776 |
| *Lgsn* | 0.738982673 | *Dbx2* | -0.729719841 |
| *Rps6ka3* | 0.736607361 | *Ckmt1* | -0.729718009 |
| *Lce3f* | 0.733114557 | *Nlrp10* | -0.713426002 |
| *Csprs* | 0.732672125 | *Dmkn* | -0.708521397 |
| *Gm2a* | 0.730180262 | *Ccdc154* | -0.701382056 |
| *Nudt6* | 0.724438348 | *Glipr1* | -0.69896487 |
| *Xlr4a* | 0.721533686 | *Cdhr1* | -0.693080235 |
| *Igf2* | 0.719016753 | *Snrnp35* | -0.690744084 |
| *Psme2b* | 0.718238516 | *Pik3ca* | -0.689792531 |
| *Phf11d* | 0.710803196 | *Ptgr1* | -0.687555556 |
| *Fcna* | 0.70628314 | *Chst5* | -0.682861878 |
| *Ccrl2* | 0.702562497 | *Npy* | -0.679370395 |
| *Il4i1* | 0.696040994 | *Gkn1* | -0.669845659 |
| *Pgm5* | 0.695860714 | *Cyp2s1* | -0.667641883 |
| *Ifi204* | 0.686940887 | *Gcat* | -0.661672203 |
| *Agfg2* | 0.674254817 | *Ung* | -0.66133132 |
| *Fbxo2* | 0.674146162 | *Sod3* | -0.661056899 |
| *Milr1* | 0.672717586 | *Ptn* | -0.657565872 |
| *Irf7* | 0.669735119 | *Gsta3* | -0.656310263 |
| *Herc6* | 0.656433159 | *Slc2a1* | -0.654268617 |
| *Dhx58* | 0.647361417 | *Calm4* | -0.649419731 |
| *Fip1l1* | 0.644689863 | *Clstn2* | -0.648274945 |
| *Cbr2* | 0.641965279 | *4931428F04Rik* | -0.646732694 |
| *AC168977.2* | 0.63701293 | *Vkorc1* | -0.637439643 |
| *Lamb3* | 0.633732074 | *Smtnl2* | -0.628405187 |
| *Ppp3cc* | 0.633067562 | *Camk2n2* | -0.626623787 |
| *Gm42674* | 0.628614435 | *1190002N15Rik* | -0.617568265 |
| *Srp14* | 0.628603336 | *mt-Nd2* | -0.614384663 |
| *Ifi44* | 0.625184148 | *Aqp2* | -0.61079724 |
| *Mpeg1* | 0.616379656 | *Myadm* | -0.596236486 |
| *Gemin7* | 0.612425427 | *Epgn* | -0.592175727 |
| *Prkg2* | 0.604152219 | *Tubb2b* | -0.591702517 |
| *Asprv1* | 0.603210257 | *Ska1* | -0.587333442 |
| *Ackr3* | 0.600985435 | *St8sia6* | -0.585942767 |
| *Medag* | 0.589078393 |  |  |
| *Psca* | 0.587510521 |  |  |
